# Chile’s 2014 sugar-sweetened beverage tax and changes in prices and purchases of sugar-sweetened beverages: An observational study in an urban environment

**DOI:** 10.1371/journal.pmed.1002597

**Published:** 2018-07-03

**Authors:** Juan Carlos Caro, Camila Corvalán, Marcela Reyes, Andres Silva, Barry Popkin, Lindsey Smith Taillie

**Affiliations:** 1 Instituto de Nutrición y Tecnología de Alimentos, Universidad de Chile, Santiago, Chile; 2 Department of Health Policy and Management, Gillings School of Global Public Health, University of North Carolina, Chapel Hill, North Carolina, United States of America; 3 Facultad de Ciencias Económicas y Negocios, Universidad Central de Chile, Santiago, Chile; 4 Carolina Population Center and Department of Nutrition, Gillings School of Global Public Health, University of North Carolina, Chapel Hill, North Carolina, United States of America; University of Cambridge, UNITED KINGDOM

## Abstract

**Background:**

On October 1, 2014, the Chilean government modified its previous sugar-sweetened beverage (SSB) tax, increasing the tax rate from 13% to 18% on industrialized beverages with high levels of sugar (H-SSBs) (greater than 6.25 grams [g] sugar/100 milliliters [mL]) and decreasing the tax rate from 13% to 10% on industrialized beverages with low or no sugar (L-SSBs) (less than 6.25 g sugar/100 mL). This study examines changes in beverage prices and household beverage purchases following the implementation of the tax reform.

**Methods and findings:**

We used longitudinal data collected between January 1, 2013, and December 31, 2015, from 2,000 households. We defined the pretax period as January 1, 2013, to September 30, 2014, and the posttax period as October 1, 2014, to December 31, 2015. We conducted a pre–post analysis for changes in prices and purchases, with the latter examined by volume and calories. We compared posttax changes in prices and purchases to a counterfactual, defined as what would have been expected in the posttax period based on pretax trends. All results are stated as comparisons to this counterfactual. We linked beverages at the bar code level to nutrition facts panel data collected by a team of Chilean nutritionists who categorized them by taxation level and beverage subcategory, which included carbonated and noncarbonated H-SSBs and concentrated, ready-to-drink L-SSBs and untaxed beverages. We reconstituted concentrated beverages and analyzed all beverages using as-consumed volumes and calories. Posttax monthly prices of H-SSBs increased, but these changes were small. Prices of carbonated H-SSBs increased by 2.0% (95% confidence interval [CI] 1.0%–3.0%), while those of noncarbonated H-SSBs increased by 3.9% (95% CI 1.6%–6.2%). Prices of L-SSB concentrates decreased after the tax by 6.7% (95% CI −8.2%–−4.6%), and prices of ready-to-drink L-SSBs increased by 1.5% (95% CI 0.3%–2.7%). Households decreased monthly per capita purchases of H-SSBs by 3.4% by volume (95% CI −5.9%–−0.9%) and 4.0% by calories (95% CI −6.3%–−1.9%), and this change was greater among high socioeconomic status (SES) households. The volume of household purchases of L-SSBs increased 10.7% (95% CI 7.5%–13.9%), while that of untaxed beverage purchases decreased by 3.1% (95% CI −5.1%–−1.1%). The main limitation of this study was that there was no control group, so we were unable to assess the causal impact of the tax.

**Conclusions:**

The modifications of Chile’s SSB tax were small, and observed changes in prices and purchases of beverages after the tax were also small. Our results are consistent with previous evidence indicating that small increases in SSB taxes are unlikely to promote large enough changes in SSB purchases to reduce obesity and noncommunicable diseases (NCDs).

## Abstract 2

Lindsey Smith Taillie and colleagues describe reduced purchases of sugar-sweetened beverages after modifications to the Chilean sugar tax.

## Author summary

**Why was this study done?:**

- Sugar-sweetened beverage (SSB) taxes have emerged as a strategy to prevent a continued rise in obesity prevalence and noncommunicable diseases (NCDs).
- Recent studies in Mexico and the United States have shown that SSB taxes are associated with increased prices of taxed beverages and reduced purchases of those beverages, particularly among households of low socioeconomic status (SES).
- Little evidence exists on the effects increasing or decreasing an existing SSB tax has on beverage prices and purchases.
- On October 1, 2014, Chile increased its tax on industrialized beverages with high levels of sugar (H-SSBs) from 13% to 18% and reduced its tax on industrialized beverages with low or no sugar (L-SSBs) from 13% to 10%. Beverages such as plain and flavored milk, 100% fruit juices, and unflavored water remained untaxed.
- Understanding the effects of changes to existing SSB taxes is critical for informing future SSB tax policy.

**What did the researchers do and find?:**

- We used a pre–post design to estimate changes in prices and purchases (volumes and calories) of beverages in Chile after the October 1, 2014, modifications of SSB taxes with data collected from January 1, 2013, to December 31, 2015. We compared observed posttax changes in beverage prices and purchases to the counterfactual, or what would have been expected in the posttax period based on pretax trends. All results are stated in terms of a comparison to this counterfactual.
- Our model links household beverage purchase data to nutrition facts panel data using bar code information to classify each beverage purchased by tax level and subcategory.
- We found that after the tax increase on H-SSBs, prices of H-SSBs increased, although the increases were small. In contrast, after the tax decrease on L-SSBs, prices decreased in some categories but increased in others. Prices of untaxed beverages also increased.
- After the tax increase on H-SSBs, purchases of H-SSBs decreased, although the declines were small. After the tax decrease on L-SSBs, purchases of L-SSBs increased. Despite no change in the tax rate, purchases of untaxed beverages decreased.
- High-SES households showed larger declines in purchases of H-SSBs after the tax than did low-SES households.

**What do these findings mean?:**

- After a small increase in the tax rate on H-SSBs, changes in prices and purchases of these beverages were small. This is consistent with previous evidence indicating that small increases in SSB taxes are unlikely to produce large changes in SSB purchases. Further evidence is needed to understand the small change in prices due to the tax reform.
- High-SES households showed larger declines in purchases of H-SSBs than did low-SES households. Further research is needed to understand the differential response to this tax by SES.
- After a decrease in the tax rate on L-SSBs, purchases of these beverages increased a large amount. Further research is needed to understand the health consequences of the shift toward these beverages, which frequently contain artificial sweeteners.

## Introduction

In response to the increasing global burden of obesity and related chronic diseases in the last decade, taxes on industrially produced sugar-sweetened beverages (SSBs) have emerged as a regulatory strategy to prevent the continued rise of obesity [1,2]. Research has shown that raising the prices of SSBs leads to significant decreases in SSB purchases [3–6], and recent studies in Mexico and cities in the US (e.g., Berkeley and Philadelphia) indicate that SSB taxes reduce purchases of SSBs, with a larger impact among lower socioeconomic status (SES) populations [7–11].

However, limited evidence exists on the impact of tax rate changes to existing SSB taxes. In high-income countries and low- and middle-income countries with rapid income growth, households might be unaware of small changes in tax rates due to higher median incomes, and commercial beverage companies might choose not to switch prices proportionally to the tax change [12]. In addition, access to safe tap water could affect choices available to consumers. For example, in Mexico, which leads in bottled water consumption worldwide [13] and has relatively limited access to safe tap water in many areas, the main substitution for SSBs in the first year after the tax implementation was water purchases [9]. In contrast, in high-income countries, free, clean water is readily available.

Chile, recently categorized as a high-income country, is an interesting case study to explore changes in prices and purchases after a change in the SSB tax rate. Chile has a high prevalence of obesity and type 2 diabetes [14–16] and recently became the country highest in SSB sales per capita [17]. Beverage taxes in Chile have a long history beginning in 1979, when the Chilean government introduced specific ad valorem taxes on alcoholic and nonalcoholic industrialized beverages. Beverage concentrates and all ready-to-drink industrialized beverages with any artificial flavoring, sweeteners, or dyes were subject to a common 15% tax rate. In 1985, the tax rate was cut to 13%. Roughly 30 years later, Chile modified its beverage tax again as part of a major tax reform that was announced and introduced in April 2014, approved in September 26, 2014, and implemented on October 1, 2014. This tax reform included an increase in the SSB tax rate that was intended to reduce purchases of SSBs and prevent continued increases in obesity and related noncommunicable diseases (NCDs). As a result, the tax rate on SSBs with greater than 6.25 grams (g) of sugar per 100 milliliters (mL) (e.g., sodas, industrialized juice drinks) increased from 13% to 18%. For SSBs with less than 6.25 g sugar per 100 mL (including powdered and concentrated beverages with added sugar and beverages containing artificial sweeteners, flavors, or dyes), the tax rate was reduced to 10%. Other beverages, such as plain milk and flavored sweetened milk-based drinks, 100% fruit juices, and unflavored water, remained untaxed.

The Chilean SSB tax structure is unique for two reasons. First, it creates a price differential between high-sugar and low-sugar SSBs, and second, it taxes beverages with artificial sweeteners (such as diet soft drinks) and flavored unsweetened beverages. In contrast, the SSB taxes in both Berkeley and Mexico apply a single rate to all nondairy and nonalcoholic beverages containing added sugar and do not apply to beverages with zero added sugar or those that contain only artificial sweeteners. The Philadelphia SSB tax is similar to Chile’s in that it also applies to artificially sweetened beverages but different in that it applies a single rate to all taxed beverages, regardless of sugar level. The United Kingdom SSB tax is also two tiered, with high-sugar SSBs taxed at a higher rate than low-sugar SSBs. However, this tax is based on the added sugar content of the beverage, and thus, artificially sweetened beverages containing no added sugar are not taxed. To our knowledge, the Chilean tax is the first that applies a two-tiered rate to SSBs and includes any artificially sweetened or flavored unsweetened beverages.

It is unclear how changing an existing SSB tax affects prices or purchases compared to the introduction of a new tax. Small sales taxes on SSBs (mean 5.2%) in jurisdictions across the US suggest that small relative increases in the tax rate may not lead to meaningful changes in sweetened beverage consumption [18,19]. However, these sales taxes are applied at the checkout and thus may not affect consumer purchasing decisions, whereas the Chilean SSB tax is included in the shelf prices of beverages. Understanding how relatively small increases in an SSB tax affect prices and purchases is critical to inform future SSB taxation policy.

The objectives of this study are to (1) analyze whether the average prices of beverage purchases changed after the tax implementation and (2) analyze whether the volumes and calories from beverage purchases changed after the tax implementation, controlling for household covariates and secular trends overall and by SES.

## Methods

### Data set

We used a longitudinal data set of household food purchases from January 2013 to December 2015 that we obtained from Kantar WorldPanel Chile (see the STROBE Checklist in S1 Table). The data are based on weekly purchases of fast-moving consumer goods by households from cities with more than 20,000 inhabitants, representing 74% of the urban population. The total sample is 2,000 households. Interviewers visited households weekly to collect data on food purchases using a handheld bar code scanner. First, information on purchases was collected either by scanning products’ bar codes on the packages or by using a codebook to assign bar codes tor bulk products or other products without bar codes. Second, households were instructed to keep all receipts so that interviewers could match purchases each week and determine the store where they were purchased (specifically for frequently consumed products). Finally, interviewers checked household pantries, and households stored empty product packages in a bin between interviews to ensure that products were not counted twice. Information collected for each beverage purchase included volume or weight, bar code expenditure, price per unit, retail channel, brand, package size, and date of purchase. A comparative analysis using the 2011–2012 Household Budget and Expenditure survey showed that households in the Kantar WorldPanel represent the average purchases of urban households [20].

#### Nutrient profile information and tax categorization

The data on each beverage purchase were linked at the bar code level to a nutrition facts panel gathered from photographs that a team of Chilean nutrition research assistants collected directly at stores (79.8% of products) [21–26], Mintel Latin America (19.9%), or Mintel North America (0.2%) or imputed with a systematic match based on similar products using package description, brand, and manufacturer (less than 0.1% of each beverage category). Master’s-level Chilean nutritionists employed by the University of North Carolina categorized each beverage purchase into mutually exclusive categories within each tax group. Hereinafter we refer to any industrialized beverages with less than 6.25 g sugar/100 mL (and therefore subject to the 10% tax rate) as low- or no-sugar sweetened beverages (L-SSBs). We refer to industrialized beverages with greater than 6.25 g sugar/100 mL (subject to the 18% tax rate) as high-sugar sweetened beverages (H-SSBs). All beverages exempted from the tax we classify as untaxed. We further separated L-SSBs based on whether they were sold as concentrated (including powders) or ready to drink. We reconstituted products purchased in powder or concentrated form to their ready-to-drink volumes and calories. All H-SSBs were ready to drink, and we categorized them by carbonated or not. We consulted Ministry of Finance staff on categorizations in cases where the appropriate category was unclear.

The final categorization within each tax group follows. Untaxed beverages: plain water, plain and flavored or sweetened milk, dairy-based ready-to-drink beverages, milk powders and modifiers (such as cocoa), coffee, and tea. Ten percent–taxed beverages: ready-to-drink L-SSBs and L-SSB concentrates. Eighteen percent–taxed beverages: noncarbonated H-SSBs and carbonated H-SSBs (S2 Table). While we analyzed untaxed beverages as a single group due to the low frequency of purchases among some subcategories, we analyzed beverages taxed at 10% and 18% by subcategory and by overall taxation level. As noted, all taxed categories were subject to a 13% tax rate prior to October 2014.

#### Socioeconomic covariates

Following the protocol of the Chilean Association of Market Research, we categorized households into four SES categories using the data Kantar WorldPanel Chile provided [27]. For simplicity, we report results of all analyses by SES in two groups: high SES (high and midhigh) and low SES (midlow and low). Other relevant household characteristics include household composition (total number of household members overall and numbers by gender and age groups), household head’s education level and working status, and household geographic region (divided into six zones based on the Kantar sample design).

In addition, because trends in income, economic activity, and other market factors could influence the price and quantity of beverage purchases [28], we controlled for several macroeconomic measures at the region-month level, including unemployment rate, population (in thousands), construction permits (meters^2^) as a predictor of economic activity [29], the Supermarket Sales Index, and the Regional Economic Index, using official figures from the Chilean National Institute of Statistics [30].

### Price indexes

For the price analysis, we sorted brands within each beverage subcategory based on their average market shares (i.e., the monthly average proportion of total sales) during the 2013–2015 period. We aggregated brands with market shares lower than 3% into a combined brand within each category. Brands with more than a 3% market share remained separate. We then classified products by market share as either low (less than or equal to 10% market share) or high (greater than 10% market share). Next, we defined package sizes for each individual brand. Ready-to-drink beverages we categorized as small (less than 2,500 mL) or large (greater than or equal to 2,500 mL). Beverages sold as liquid concentrates, powders, or dry leaves we categorized as small (less than 5 liters [L], diluted) or large (greater than or equal to 5 L, diluted). Hereinafter we refer to a product as the unique combination of brand and package size within each beverage category.

### Market price aggregation

Our analysis used data on beverage prices as reported directly by households. Unlike data on prices collected from food stores, prices obtained from purchase data not only reflect changes in prices due to industry behavior but also differences in household preferences for certain beverage types or geographic differences in what products are available in the store (e.g., local brands may be available in some regions but not in others) [31]. In other words, the only prices captured are for beverages that are purchased, making it difficult to analyze whether the industry changed shelf prices in response to the tax modification. To deal with this complexity, we attempted to approximate prices consumers would see in a store by exploiting the geographic and socioeconomic variations across households. We defined a market as the pool of unique purchases that belong to the same region and SES group in the same month (using the product classifications described above). Thus, the average product-level prices in each market reflect the average price in a month for a given product that a household of a given SES and region would be likely to see in a store. We excluded from the analysis products that were not purchased both before and after the tax implementation (0.4% of all observations).

### Descriptive statistics

First, we examined sociodemographic characteristics of the households participating in the survey by SES status and separated households that exited the sample at any point between 2013 and 2015. Next, we calculated the average and median adjusted real prices at the market-month level, including the number of products within each beverage category. Finally, we calculated average purchases by household/month both by volume (mL) and calories (kilocalories [kcal]) purchased per capita per day for each beverage category and the percentage of nonzero household/month observations.

### Analysis plan

We conceived the initial study design in early July 2016 and purchased the Kantar data at the end of the same month. Between July 2016 and May 2017, master’s-level nutritionists cleaned and categorized the data, and we conducted descriptive analyses on volumes of purchases. Prior to any analysis, the initial analysis plan included:

- Descriptive unadjusted trends in volume, sugar, and calories by taxation category and beverage subcategories and product characteristics.
- Descriptive unadjusted trends in volume, sugar, and calories by taxation category for each SES subgroup.
- Longitudinal analysis adjusted for household and macroeconomic covariates using fixed effects models for volume, sugar, and calories by taxation status and beverage subcategory. Pretax trends were to be projected into the posttax period to serve as a counterfactual for observed posttax purchases, with the statistical tests conducted between the observed and the counterfactual posttax average household purchases. This followed the framework laid out in previous studies of the Mexican SSB tax [9].
- Using the same longitudinal models, stratify by SES to examine whether there were differences between low- and high-SES households.

In July 2017, we introduced two changes to the original analysis plan. First, we realized that, unlike in the Mexican tax evaluation, in Chile, no studies had examined changes in prices due to the tax implementation. Understanding changes in prices seemed critical to explaining any changes in beverage purchases (or lack thereof) after the SSB tax. Thus, we added a price analysis component, which included analysis of how estimated changes vary by product characteristics and market-level SES. Second, for both analyses, we tested several models to determine the best model specification for our data. As a result, we used a correlated random effects tobit model for volumes and a linear random effects model for prices. However, despite these modifications, the goal of the analysis remained the same: to compare observed posttax changes in prices and purchases to a counterfactual, or what would have been observed in the posttax period as predicted by pretax trends.

Finally, we introduced a few additional changes as requested during the review process, including the addition of 95% confidence intervals (CIs), sensitivity analyses on model specification, and reorganization of the tables in the main text. As requested, we also included the statistical significance of our results accounting for multiple hypotheses testing.

### Pre–post analysis framework

After a preliminary and descriptive analysis of the data set, we developed a pre–post analysis model to address the lack of a proper control group, since the tax was implemented at a national level. The goal of this analysis is to capitalize on changes in the average mean that occur before and after an intervention based on a break in time (the date of tax implementation). This approach is consistent with previous studies of Mexico’s SSB tax [9,10,32]. In this framework, for each analysis, we constructed a counterfactual, which represents the average predicted change in an outcome variable in the posttax period based on pretax trends. For each outcome, we calculated the difference between the observed trend and the counterfactual, on average, during the posttax period. Thus, each analysis is stated in terms of a comparison of what was observed after the tax compared to what might have been observed without a tax based on pretax trends (which is similar to an interrupted time series design). To ensure that our counterfactual captured the effect of the intervention rather than other contemporaneous changes, we controlled for time-varying confounders, seasonality, and national and regional trends, explained in more detail in the purchase analyses section. We calculated the statistical significance for each estimate adjusting for multiple hypotheses testing using the Sidak–Dunn correction [33]. Additionally, we reported nonconservative 95% CIs for each estimate. We estimated all models using Stata v.14.1.

### Price analyses: Random effects model

We conducted all analyses at the product-market level using the natural logarithm of prices (assuming that prices follow a log-normal distribution). The unit of each observation is the price of each product per 1,000 mL in a market in a given month. We used a linear random effects model with standard errors clustered at the market level. This model uses a tax period indicator variable (pretax versus posttax) to create a break in the average mean purchases before and after the tax implementation. We controlled for linear and quadratic aggregated trends, seasonality, and regional economic covariates. Additionally, we examined whether price changes vary by low versus high market-level SES, package size, and the brand’s market share using interactions with the tax indicator variable.

After estimating the model, we calculated the adjusted predicted changes in price level and its corresponding 95% CI, comparing what was observed in the posttax period to the counterfactual (with the tax indicator variable set to zero). To obtain the predicted values, we back-transformed the conditional means, applying Duan smearing factors [34].

### Purchase analyses: Correlated random effects tobit model

For our purchases analysis, the unit of observation was monthly per capita volumes or calories of beverages purchased by the household. We used a tobit model to explicitly recognize the large proportion of nonpurchases of each subcategory and to minimize biases introduced by the large proportion of nonpurchasers. We clustered standard errors at the household level.

Additionally, to adjust for unobserved heterogeneity by household (such as underlying differences in household preferences), we allowed for correlated random effects at the household level using the Chamberlain–Mundlak device [35,36]. We also included time-varying controls: household size, household composition by gender and age group, head of household education (no formal education, middle school, high school, college or more) and working status (unemployed, working, studying), indicator variables for region and SES (low and midlow versus midhigh and high), linear and quadratic aggregated trends, and quarterly seasonal indicator variables. We interacted SES with the tax indicator variable to analyze changes in effects by SES. We excluded from the calories analysis the beverage categories with no or very low sugar content (10% tax rate beverages), since the calorie content of these beverages is too low to analyze differences.

### Sensitivity analyses

We conducted several sensitivity analyses to determine the best model specification using information criterion and goodness of fit for model selection. First, we tested different specifications for the trends, time breaks, and seasonality in each model and the significance of the time-varying covariates in each specification. Second, we tested the sensitivity of our estimates to autocorrelation (i.e., the similarity of the observations of each household over time) using the Arellano–Bond estimator [37]. We also estimated a static hurdle model to relax the assumptions imposed by the tobit model (i.e., that covariates have the same effect on both the probability of purchase and the amount purchased). Finally, as noted above, we examined interactions of the tax indicator with market-level SES, package size, and brand size for the price analyses and household-level SES for the purchase analyses. For each of these subgroup analyses, we report the *p*-value of a *t* test on whether the difference in the absolute predicted mean changes between the two groups in the posttax period is statistically different from zero.

## Results

### Descriptive statistics

Eighty-five percent of households were in the sample for all 36 months. Our analytic sample includes 1,795 unique households, 64,620 household monthly observations, and 114,003 market-month product observations.

Household characteristics in our sample are summarized in S3 Table. In this sample, low-SES households, compared to high-SES households, were more likely to live in Santiago and have an employed household head, a larger overall household size (including more children but fewer adults), and less education. Households were more likely to leave the survey if the household head was younger and slightly more educated, although we found no significant differences in household composition or SES. Given this, our estimates were slightly biased toward older households, conditional on being surveyed in January 2013.

Unadjusted average monthly market prices by beverage subcategory showed major differences between subcategories (S4 Table). We found the highest prices for noncarbonated H-SSBs. L-SSB concentrates were the least-expensive beverage type. We found the smallest (relative) differences in prices across SES among carbonated H-SSBs.

S5 Table shows average purchases (in volume and calories) at the household-month level. Carbonated H-SSBs (soft drinks) and untaxed beverages were purchased most frequently. Purchases of H-SSBs represent 18.0% of the total beverage purchases by volume and 55.0% of the total beverage purchases by calories in our data. Purchases of L-SSB concentrates were relatively large (10.0% of total beverage purchases) but contributed to a minimal proportion of calories purchased (0.2%). High-SES households purchased more of all beverage categories compared to low-SES households except for carbonated H-SSBs (2.3 versus 2.8 L/capita/month for high-SES and low-SES households, respectively).

The distribution of package size and brand size (i.e., market share) for all taxed products (weighted by total sales) are in S1 and S2 Figs, respectively. These results confirm that the use of 10% as a cutoff to define high versus low market share and the use of 2,500 mL to define small versus large package size was appropriate.

Monthly adjusted mean prices and purchases (volume) by beverage category are in S3 and S4 Figs, respectively. We found no obvious changes in prices or purchases after tax implementation, with one exception: the price of L-SSB concentrates decreased significantly following tax implementation. Finally, we tested normality in the distribution of prices (in logs) using the Shapiro–Wilk test on a random sample of products (S6 Table) and found that this assumption cannot be rejected.

### Price analysis

Table 1 shows changes in average adjusted real prices at the product level (actual versus counterfactual estimates) by subcategory and tax regime by SES market and overall. Compared to the counterfactual, prices of carbonated H-SSBs rose by 2.0% (95% CI 1.0%–3.0%), and prices of noncarbonated H-SSBs rose by 3.9% (95% CI 1.6%–6.2%).

**Table 1:** Average adjusted real prices, actual versus counterfactual estimates (Chilean pesos).

Category	Absolute difference			Percentage change			High versus low SES (*p*-value)
	Low SES	High SES	Overall	Low SES	High SES	Overall	
*Untaxed*	**22.8 (14.0, 31.7)**	1.5 (−7.6, 10.7)	**13.0 (5.0, 21.1)**	**3.2% (1.9%, 4.5%)**	0.2% (−1.0%, 1.4%)	**1.8% (0.7%, 2.9%)**	0.000
*Taxed 10%*							
Ready-to-drink L-SSBs	**14.5 (5.1, 23.9)**	7.5 (−4.5, 19.4)	11.1 (2.2, 20.0)	**2.0% (0.7%, 3.3%)**	0.9% (−0.5%, 2.3%)	1.5% (0.3%, 2.7%)	0.161
L-SSB concentrates	−**12.1 (**−**18.6**, −**5.6)**	−**10.3 (**−**15.2**, −**5.6)**	−**11.0 (**−**13.6**, −**7.6)**	−**7.6% (**−**9.8%**, −**3.5%)**	−**5.8% (**−**8.6%**, −**3.2%)**	−**6.7% (**−**8.2%**, −**4.6%)**	0.548
*Taxed 18%*							
Noncarbonated H-SSBs	**27.7 (10.1, 45.3)**	**39.1 (15.1, 63.1)**	**33.3 (13.9, 52.7)**	**3.3% (1.2%, 5.4%)**	**4.4% (1.7%, 7.1%)**	**3.9% (1.6%, 6.2%)**	0.254
Carbonated H-SSBs	**14.7 (7.5, 21.9)**	**15.1 (6.4, 23.8)**	**15.1 (6.4, 24.0)**	**2.0% (0.9%, 3.2%)**	**1.9% (0.8%, 3.0%)**	**2.0% (1.0%, 3.0%)**	0.894

Note: 95% CIs in parentheses. Results significantly different from zero indicated in bold, using the Sidak–Dunn method for multiple hypotheses testing (critical value alpha = 0.0102). Unweighted estimates. The *p*-value for high versus low SES is for the difference in the absolute predicted means between the two groups in the posttax period. Observations in each model as indicated in S4 Table.

Abbreviations: CI, confidence interval; H-SSB, industrialized beverage with high levels of sugar; L-SSB, industrialized beverage with low or no sugar; SES, socioeconomic status.

Compared to the counterfactual, L-SSB concentrates saw a sizable price reduction following the tax cut (−6.7%, 95% CI −8.2%–−4.6%), while prices of ready-to-drink L-SSBs increased slightly (1.5%, 95% CI 0.3%–2.7%). After the tax, the prices of untaxed beverages increased by 1.8% compared to the counterfactual (95% CI 0.7%–2.9%).

We found no significant differences in price changes for H-SSBs or L-SSBs by SES market. However, among untaxed beverages we found a significant increase in price for the low-SES market but no change in price for the high-SES market (*p*-value = 0.000).

S7 Table shows changes in average adjusted real prices at the product level (actual versus counterfactual estimates) by package size and market share. Overall, we found larger price variations in small packages relative to large packages (particularly for L-SSB concentrates). In terms of market share, we noted significant differences only in the price changes among L-SSB concentrates (*p*-value = 0.000). While L-SSBs with large market shares increased prices (despite the cut in the tax rate), brands with small market shares experienced a significant decrease in prices.

### Purchase analysis

Absolute and relative changes in household beverage purchases by volume and calories are in Tables 2 and 3, respectively. Compared to the counterfactual, posttax household purchases of H-SSBs decreased in both volume (−3.4%, 95% CI −5.9%–−0.9%) and calories (−4.0%, 95% CI −6.3%–−1.9%). At the subcategory level, carbonated H-SSB purchases did not decrease by volume but did decrease 3.0% by calories (95% CI −5.2%–−0.8%) relative to the counterfactual. Noncarbonated H-SSBs decreased 8.2% by volume (95% CI −13.6%–−3.0%) and 8.9% by calories (95% CI −13.6%–−4.2%) relative to the counterfactual.

**Table 2:** Household average monthly purchased volume, actual versus counterfactual estimates (mL/person/month).

Category	Absolute difference			Percentage change			High versus low SES (*p*-value)
	Low SES	High SES	Overall	Low SES	High SES	Overall	
*Untaxed*	−**472 (**−**723**, −**221)**	−213 (−497, 71)	−**371 (**−**611**, −**131)**	−**4.3% (**−**6.6%**, −**2.0%)**	1.4% (−3.3%, 0.5%)	−**3.1% (**−**5.1%**, −**1.1%)**	0.035
*Taxed 10%*	**223 (136, 311)**	**373 (271, 475)**	**281 (197, 366)**	**9.5% (5.8%, 13.2%)**	**10.8% (7.8%, 13.8%)**	**10.7% (7.5%, 13.9%)**	0.006
Ready-to-drink L-SSBs	**75 (39, 111)**	**204 (150, 258)**	**123 (84, 162)**	**12.2% (6.3%, 18.1%)**	**14.3% (10.5%, 18.1%)**	**12.3% (8.4%, 16.2%)**	0.001
L-SSB concentrates	**186 (120, 252)**	**129 (63, 198)**	**165 (105, 225)**	**10.7% (6.9%, 14.5%)**	**7.1% (3.5%, 10.9%)**	**9.4% (6.0%, 12.8%)**	0.103
*Taxed 18%*	−53 (−139, 33)	−**194 (**−**283**, −**105)**	−**108 (**−**187**, −**28)**	−1.6% (−4.2%, 1.0%)	−**6.4 (**−**9.3%**, −**3.5%)**	−**3.4% (**−**5.9%**, −**0.9%)**	0.004
Noncarbonated H-SSBs	−**45 (**−**71**, −**18)**	−37 (−70, −3)	−**41 (**−**68**, −**15)**	−**10.1% (**−**16.3%**, −**4.1%)**	−5.3% (−10.0%, −0.4%)	−**8.2% (**−**13.6%**, −**3.0%)**	0.656
Carbonated H-SSBs	−8 (−88, 72)	−**167 (**−**246**, −**88)**	−70 (−142, 2)	−0.2% (−2.2%, 1.8%)	−**7.2% (**−**10.6%**, −**3.8%)**	−2.6% (−5.3%, 0.1%)	0.001

Note: Analytic sample considers only households present in the sample during the complete period. 95% CIs in parentheses. Results significantly different from zero indicated in bold, using the Sidak–Dunn method for multiple hypotheses testing (critical value alpha = 0.0102). Unweighted estimates. The *p*-value for high versus low SES is for the difference in the absolute predicted means between the two groups in the posttax period. 64,620 household monthly observations.

Abbreviations: CI, confidence interval; H-SSB, industrialized beverage with high levels of sugar; L-SSB, industrialized beverage with low or no sugar; SES, socioeconomic status.

**Table 3:** Household average monthly purchased calories, actual versus counterfactual estimates (kcal/person/month).

Category	Absolute difference			Percentage change			High versus low SES (*p*-value)
	Low SES	High SES	Overall	Low SES	High SES	Overall	
*Untaxed*	−**44 (**−**73**, −**15)**	−**71 (**−**106**, −**36)**	−**54 (**−**83**, −**25)**	−**3.0% (**−**5.0%**, −**1.0%)**	−**7.6 (**−**11.3%**, −**3.9%)**	−**5.3% (**−**8.1%**, −**2.5%)**	0.091
*Taxed 18%*	−37 (−68, −7)	−**81 (**−**113**, −**49)**	−**55 (**−**83**, −**26)**	−2.6% (−4.8%, −0.5%)	−**6.5% (**−**9.1%**, −**3.9%)**	−**4.0% (**−**6.3%**, −**1.9%)**	0.006
Noncarbonated H-SSBs	−**22 (**−**34**, −**11)**	−18 (−32, −4)	−**21 (**−**32**, −**10)**	−**10.4% (**−**16.1%**, −**5.2%)**	−5.8% (−10.3%, −1.3%)	−**8.9% (**−**13.6%**, −**4.2%)**	0.505
Carbonated H-SSBs	−13 (−41, 15)	−**67 (**−**94**, −**39)**	−**34 (**−**59**, −**9)**	−1.1% (−3.5%, 1.3%)	−**7.2% (**−**10.1%**, −**4.2%)**	−**3.0% (**−**5.2%**, −**0.8%)**	0.000

Note: Analytic sample considers only households present in the sample during the complete period. 95% CIs in parentheses. Results significantly different from zero indicated in bold, using the Sidak–Dunn method for multiple hypotheses testing (critical value alpha = 0.0127). Unweighted estimates. The *p*-value for high versus low SES is for the difference in the absolute predicted means between the two groups in the posttax period. 64,620 household monthly observations.

Abbreviations: CI, confidence interval; H-SSB, industrialized beverage with high levels of sugar; SES, socioeconomic status.

In contrast, the volume of household purchases of L-SSBs increased 10.7% (95% CI 7.5%–13.9%) relative to the counterfactual. By subcategory, we observed a 9.4% increase in purchase volume of L-SSB concentrates (95% CI 6.0%–12.8%) and a 12.3% increase in ready-to-drink L-SSBs (95% CI 8.4%–16.2%) relative to the counterfactual. Purchases of untaxed beverages decreased 3.1% by volume (95% CI −5.1%–−1.1%) and 5.3% by calories (95% CI −8.1%–−2.5%).

We found that posttax changes in H-SSB purchases varied by household SES (*p*-value = 0.004 for volume and 0.006 for calories). In general, high-SES households showed larger declines in H-SSB purchases after the tax than did low-SES households. Specifically, compared to their respective counterfactuals, high-SES households reduced purchases of H-SSBs by 6.4% by volume (95% CI −9.3%–−3.5%) and 6.5% by calories (95% CI −9.1%–−3.9%), whereas low-SES households showed no change in the volume or calories of H-SSB purchases.

We also found that posttax changes in the volume of L-SSB purchases varied by household SES (*p*-value = 0.006), though the size of the difference between SES groups was small. Specifically, relative to their respective counterfactuals, high-SES households increased the volume of L-SSB purchases by 10.8% (95% CI 7.8%–13.8%), while low-SES households increased the volume of L-SSB purchases by 9.5% (95% CI 5.8%–13.2%). Finally, there were also statistically significant differences in posttax changes of untaxed beverage purchases in high- versus low-SES households by volume (*p*-value = 0.035) but not by calories (*p*-value = 0.091). Specifically, relative to their respective counterfactuals, high-SES households had no change in the volume of untaxed beverages purchased, while low-SES households decreased the volume of untaxed beverage purchases by 4.3% (95% CI −6.6%–−2.0%).

### Sensitivity analysis

The results presented in this paper reflect the best model fit based on Akaike information criteria (AIC) [38] and goodness of fit (R-squared), taking into account the particular characteristics of each model. A summary of results of different model specifications and AIC for selected outcomes is in S8 Table. We note that alternative model specifications provide less precise results compared to our chosen model specification, although results follow similar patterns. The model with a break in trends and intercepts provides more precise estimates for some outcomes. However, in this model, there are no significant differences in the pretax and posttax trend estimates; thus, results are to be interpreted with caution. For analyses of the volume of purchases, we found that a static hurdle model provided similar though less precise results due to an inability to capture household unobserved time-invariant heterogeneity (in household preferences, for example). Finally, we noted the lack of significance of the pre–post indicator when we switched it to April 2014, when the law was introduced in the National Congress of Chile, which is suggestive evidence that there was no significant anticipatory behavior prior to the tax implementation.

## Discussion

### Main findings on H-SSB and L-SSB prices and purchases

In October 2014, Chile increased its tax rate on H-SSBs from 13% to 18% and reduced its tax rate on L-SSBs from 13% to 10%, representing an 8% spread between L-SSBs and H-SSBs after tax implementation. This study found that after the 5% tax increase on H-SSBs, prices increased by 2.0% for carbonated H-SSBs and 3.9% for noncarbonated H-SSBs relative to their respective counterfactuals. After the 3% tax decrease on L-SSBs, price changes were heterogeneous, with prices decreasing for concentrated L-SSBs by 6.7% and for ready-to-drink L-SSBs by 1.5% relative to their respective counterfactuals.

Commensurate with the small increase in prices of H-SSBs after the tax relative to the counterfactual, we found a small decrease in household purchases of H-SSBs (−3.4% by volume and −4.0% by calories), with most of the declines coming from noncarbonated H-SSBs (−8.2%). Overall household purchases of L-SSBs increased relative to the counterfactual (10.7% for volume), with larger changes among ready-to-drink L-SSBs (14.3%).

### Changes in prices of L-SSBs and H-SSBs after the tax

The price increases of H-SSBs were small (2.0% for carbonated and 3.9% for noncarbonated H-SSBs relative to the respective counterfactuals) compared to the tax increase of 5% on these beverages. This is different from what was observed in Mexico, where shelf prices of carbonated SSBs increased proportionally to the size of the tax [32]. However, there are several important differences between this study of Chile’s tax and studies of Mexico’s tax. First, the current study examined only available prices of beverages that were purchased, whereas in Mexico and in most price analyses, the data come from the food store environment, allowing comparison of shelf prices for the same products over time. Second, changing store prices has administrative costs, which might have prevented some brands from increasing prices, especially considering the relatively small size of the tax hike in Chile (half the size of the tax implemented in Mexico). Further research is needed to determine whether the Chilean SSB tax hike led to a proportional change in shelf prices.

We also found that for H-SSBs and L-SSBs, price changes varied across beverage subcategories when we considered differences in brand size, package size, and market-level SES. While it is outside the scope of this article to explain commercial beverage company behavior, in Chile, companies commonly maintain a complex portfolio of carbonated H-SSBs and carbonated L-SSBs (e.g., regular and diet soda), which allows for a cross-subsidy between tax categories to maintain profits. This could explain the small absolute difference in the prices of ready-to-drink L-SSBs and carbonated H-SSBs after the tax. In contrast, L-SSB concentrates and noncarbonated H-SSBs tend to be manufactured by different companies, preventing them from cost-shifting from one category to another. This may explain why among these beverage types the observed price changes were consistent with the tax changes (i.e., the price of L-SSB concentrates decreased after the tax, consistent with the tax rate decrease on these beverages, while the price of noncarbonated H-SSBs increased after the tax, consistent with the tax rate increase on these beverages). Similar industry responses were observed in Mexico, where price changes varied geographically and by package size [32,39]. Future research should examine changes in beverage company behavior due to this complex tax structure.

### Changes in household purchases of H-SSBs and L-SSBs after the tax

With regard to household purchases of H-SSBs, after the tax, there was an overall decline of 3.4% by volume relative to the counterfactual. This reflected substantial heterogeneity by beverage subcategory, with an 8.2% decline in volume for noncarbonated H-SSBs and a 2.6% decline in volume for carbonated H-SSBs. Although these posttax declines in volume purchased are larger than the corresponding changes in price, these findings are consistent with previous studies of price elasticities (i.e., sensitivity to price) of SSBs in Chile, which range from −1.30 to −1.37 [4,40]. In other words, based on these elasticities, for a 2.0% increase in price, we would have expected consumers to decrease H-SSB purchases by approximately 2.6%, consistent with our results.

Despite these declines in purchases, however, this tax is likely to have a small impact on preventing excess caloric intake or adverse health effects associated with SSB intake. This is because the absolute reductions in H-SSB purchases were small. For example, compared to the counterfactual, the overall volume of purchases of H-SSBs decreased by 108 mL per person per month (equivalent to approximately a third of a can of soda). Similarly, calories purchased decreased by 3.0% and 8.9%, respectively, for carbonated H-SSBs and noncarbonated H-SSBs compared to their respective counterfactuals. This reflects absolute reductions of only 34 calories per person per month for carbonated H-SSBs and 21 calories per person per month for noncarbonated H-SSBs compared to their respective counterfactuals. To put this in context, Mexico’s SSB tax was about 10%, which translated to a 6% decline in volume of purchases relative to the counterfactual in the first year after tax implementation but represented a larger absolute decline of 360 mL per person per month compared to the counterfactual (with no results yet on corresponding calorie changes) [9]. In Chile, these small changes in volume and calories purchased seem unlikely to have large effects on obesity and NCD risk, but more research is needed to understand any potential implications for dietary intake and weight gain.

On the contrary, purchases of L-SSBs increased considerably in the posttax period (10.7% relative to the counterfactual). This translates into absolute increases of 281 mL per person per month (or roughly three-fifths of a can of soda) beyond what would be expected based on pretax trends. We were unable to estimate changes in calories purchased for L-SSBs, because many of them had very few or no calories. While the absolute changes in volume of L-SSB purchases were small, the relative percentage increase may be concerning, particularly for high consumers, given the lack of evidence regarding the long-term health effects of consumption of artificial sweeteners [41].

There are several possibilities that explain the smaller declines in H-SSB purchases after Chile’s tax compared to Mexico’s tax. First, as noted above, the estimated relative price increases on H-SSBs were smaller in Chile than in Mexico. Second, in Mexico, the SSB tax was accompanied by a prolonged, multiyear advocacy campaign that included coalition-building across key scientific and consumer advocacy organizations and paid and earned media campaigns, public demonstrations, press conferences, scientific forums, and civil society forums [42]. Such campaigns have the potential to influence consumers’ social norms, attitudes, and purchasing behaviors independently of the regulation itself, as was shown in a decline in SSB consumption in Mexico aligned with the initiation of the public discourse [9,43]. For example, a recent study found that a public awareness campaign to decrease SSB intake via TV advertising, digital marketing, outdoor advertising, social media, and earned media was associated with an accelerated decrease in SSB purchases [44]. In Chile, the SSB tax modification was a small component of a major fiscal reform and as such lacked significant advocacy campaigns. This might have limited potential changes in social norms and attitudes related to SSB consumption.

### Changes in prices and purchases of untaxed beverages after the tax

Even though the Chilean tax modification did not affect previously untaxed beverages, such as milk or 100% fruit and vegetable juices, this study found that prices of these beverages increased 1.8% in the posttax period relative to the counterfactual. Our estimates suggest that the changes in the relative prices of taxed beverages affected the markets of untaxed products, particularly in low-SES markets (which experienced a 3.2% increase in prices of untaxed beverages relative to the counterfactual).

Commensurate with this price increase, household purchases of untaxed beverages decreased in the posttax period (−3.1% by volume and −5.3% by calories relative to the counterfactual).

Interestingly, the largest reduction in calories purchased after the tax change came from untaxed products rather than taxed H-SSBs. One possibility is that consumers substituted L-SSBs for untaxed beverages, which is consistent with the finding that after the tax decrease on L-SSBs, untaxed beverages became relatively more expensive. Because different untaxed beverage subcategories have varying amounts of sugar (such as lactose in plain milk and added sugar in sweetened flavored milk) and beneficial nutrients, like calcium in milk or vitamin C in 100% juice, it will be useful for future research on SSB taxes to examine the changes in purchases that occur across various untaxed beverage subcategories and the potential implications of these changes for health.

### Price and purchase changes after the tax by SES

This study found significant differences in changes in price after the tax by market-level SES for untaxed beverages but not for H-SSBs or L-SSBs. It was not clear why changes in the price of untaxed beverages would vary by market-level SES. We were unable to tell whether these differences in the change in price by SES market were due to differential price changes by retailers and manufacturers or differences in consumer behavior, as noted earlier. For example, if low-SES households change how often they buy products or favor bulk discounts on large products, it could lead to variations in the price changes across markets. More research is needed to explore differential price changes by SES market after the tax to understand the implications for both consumer behavior and industry behavior.

We found that high-SES households had a larger decline in the volume of H-SSB purchases (−6.4%) than did low-SES households (−1.6%) relative to their respective counterfactuals. This was mainly driven by differences in purchases of carbonated H-SSBs. While high-SES household volume purchases of H-SSBs decreased by 7.2%, low-SES household volume purchases did not change. Low-SES households showed larger declines in noncarbonated H-SSBs than high-SES households, but the difference was not statistically significant. In addition, it is important to note that carbonated H-SSBs represent a larger proportion of SSB purchases than do noncarbonated H-SSBs, so the SES differences for carbonated H-SSBs are especially important.

The result—that high-SES households had bigger changes in purchases of H-SSBs—contrasts with the Mexican first-year SSB tax evaluation, which found a 9.2% decline in low-SES households compared to a 5.6% decline for high-SES households [9]. This result is also contrary to expectations that lower-SES households will respond more to price increases due to a higher price sensitivity in this group. There are several potential explanations for these counterintuitive findings. Low-SES households could be more likely to intentionally avoid the tax by changing purchasing strategies, such as making larger but less frequent purchases [45]. Also, low-SES households in Chile are higher consumers of H-SSBs (relative to high-SES households) and thus may be less likely respond to price changes, because they have a stronger preference for these beverages [46]. While the absolute reductions in H-SSB purchases were small for all SES groups, the higher changes observed among high-SES households are concerning. Considering that SSB taxes are expected to produce larger changes in behavior among low-SES households and that this expectation is often used to rationalize the use of such taxes to reduce SES-related disparities in obesity and NCDs, future research should examine the drivers of this differential response by SES and potential implications for diet and health.

Finally, we found differences in changes in posttax purchases by household SES. Low-SES households showed larger reductions in untaxed beverage purchases by volume, but high-SES households showed larger reductions in untaxed purchases by calories. These differences in response by household SES could be the result of changes in the types of untaxed beverages purchased, since this category is very heterogenous and includes beverages with calories, such as milk and 100% juice, but also beverages with no calories, such as plain bottled water. We were unable to examine differences by beverage subcategory to better explain these findings due to the low amount of purchases in some of these subcategories.

### Strengths and limitations

The most important limitation of this study is our inability to assess a causal relationship between the tax modifications and changes in prices or purchases due to the potential presence of other simultaneous trends affecting underlying preferences and due to the inability of household food purchase data to capture all beverages consumed (particularly those consumed out of the home). While we did adjust for economic factors and secular trends, we cannot rule out shifts in preferences or norms that may have occurred concurrently with the tax implementation. To address our inability to capture all beverages using household purchase data, we will conduct future research using dietary recall data to capture the full range of beverages consumed. In the meantime, we note that the trends observed here are consistent with those in Euromonitor sales data, which reflect all beverages sold in the country [47]. In terms of external validity, we note that our sample is more likely to represent urban and older households and therefore does not completely reflect changes in purchases among younger households and the rural population. However, a comparative analysis of our data shows that the distribution of purchases by beverage group is consistent with those reported in the 2011–2012 Chilean Budget and Expenditure Survey. Furthermore, this study examines only the first posttax year in Chile. It is possible that subsequent years will see additional purchasing changes, as occurred in Mexico [10].

An additional limitation of this study is that, as previously noted, the prices reflected products purchased and not those available in stores (i.e., what was available in the market before and after the tax). However, we were able to address this issue using models that consistently captured variability in prices, allowing us to accurately estimate the average market changes while also exploring heterogeneity across product and brand characteristics. We also explicitly accounted for the censored nature of purchasing data by estimating a tobit model that predicts both the choice to purchase and the amount purchased. Finally, we were able to examine differential changes in both prices and purchases by SES (at the market level for price and at the household level for purchases). An understanding of differences in tax responses by SES is important for understanding how the tax may affect existing disparities in diet and health.

### Future research

Additional research is needed to understand the observed changes in prices and purchases and the differential responses by SES. For example, one possibility is that companies changed their marketing strategies to mitigate the effects of the price increase. A second possibility is that companies could have changed the nutritional formulation of products to avoid the tax. We also want to note that a subsequent Chilean law in July 2016 implemented front-of-package warning labels, restrictions on marketing to children, and restrictions on sales in schools for all foods and beverages containing high levels of sugar, sodium, saturated fat, or calories, which could have had additional influence on both commercial beverage industry behavior and consumer behavior in the time period leading up to the implementation of those regulations. Given the substantial regulatory changes underway in Chile’s food environment since 2014, a critical question for future research is whether this small SSB tax modification, along with the newer marketing and media controls and front-of-package labels, will result in sustained changes in Chileans’ dietary intake with potential downstream effects for SES disparities in obesity and NCDs.

Finally, given the large and increased consumption of ready-to-drink L-SSBs and L-SSB concentrates, we recommend that the effects of these beverages on health be further investigated in the Chilean population, especially considering that they are relatively cheaper than their high-sugar alternatives and often bear significantly more front-of-package marketing, such as nutrition and health claims [48].

### Conclusion

The small increase (5%) in the Chilean tax rate on H-SSBs was followed by small decreases in prices and purchases of these beverages, with high-SES households showing larger declines in purchases than low-SES households. The small decrease (3%) in the tax on L-SSBs was followed by heterogeneous changes in prices and increases in purchases of these beverages. Prices of untaxed beverages increased, and purchases of these beverage decreased, despite the lack of a tax or a change in the tax rate on these beverages. Our results are consistent with previous evidence indicating that small tax rates on SSBs are unlikely to promote the changes in SSB purchases needed to reduce obesity and NCDs.

## Supporting information

S1 Table STROBE statement—Checklist of items that should be included in reports of cohort studies. (DOC)

S2 Table Beverage categorization system. (PDF)

S3 Table Household-level descriptive statistics (household-month averages). (PDF)

S4 Table Market-level price descriptive statistics (market-month averages). (PDF)

S5 Table Household-level purchases descriptive statistics (household-month averages). (PDF)

S6 Table Shapiro–Wilk normality test of log prices for selected products. (PDF)

S7 Table Average adjusted real prices, actual versus counterfactual estimates by brand market share and package size (Chilean pesos). (PDF)

S8 Table Alternative model specifications for selected categories. (PDF)

S1 Fig Distribution of package size of taxed products (weighted by total sales). (TIF)

S2 Fig Distribution of market share of taxed products (weighted by total sales). (TIF)

S3 Fig Monthly adjusted real prices by beverage category (2015 Chilean pesos). H-SSBs and ready-to-drink L-SSBs on left axis, L-SSB concentrates on right axis. H-SSB, industrialized beverage with high levels of sugar; L-SSB, industrialized beverage with low or no sugar.
(TIF)

S4 Fig Monthly per capita purchases by beverage category (mL). (TIF)

## Abbreviations

AIC: Akaike information criteria
CI: confidence interval
H-SSB: industrialized beverage with high levels of sugar
INFORMAS: International Network for Food and Obesity/Non-Communicable Diseases Research
L-SSB: industrialized beverage with low or no sugar
NCD: noncommunicable disease
SES: socioeconomic status
SSB: sugar-sweetened beverage
