# Supplementary material for: Chile’s 2014 sugar-sweetened beverage tax and changes in prices and purchases of sugar-sweetened beverages: An observational study in an urban environment
Source: PLoS Med. 2018 Jul 3;15(7):e1002597. doi: 10.1371/journal.pmed.1002597 (PMC6029755; doi:10.1371/journal.pmed.1002597)
Supplement: S1 Table — (DOC) [file pmed.1002597.s001.doc]

**S1 Table.** STROBE Statement—Checklist of items that should be included in reports of ***cohort studies***

|  | Item No. | Recommendation |
| --- | --- | --- |
| **Title and abstract** | 1 | (*a*) Indicate the study’s design with a commonly used term in the title or the abstract [**See the title of the manuscript, “observational study in an urban environment,” and sentence 1 of the Abstract: Methods and Findings, sentence 1.]** |
|  |  | (*b*) Provide in the abstract an informative and balanced summary of what was done and what was found  **[See Abstract: Methods and Findings.]** |
| Introduction | | |
| Background/rationale | 2 | Explain the scientific background and rationale for the investigation being reported **[See Introduction, paragraphs 1–5.]** |
| Objectives | 3 | State specific objectives, including any prespecified hypotheses.  **[See Introduction, paragraph 6.]** |
| Methods | | |
| Study design | 4 | Present key elements of study design early in the paper.  **[See Methods, subheadings Data set, Price indexes, Market price aggregation, Descriptive statistics, and Pre-post analysis framework.]** |
| Setting | 5 | Describe the setting, locations, and relevant dates, including periods of recruitment, exposure, follow-up, and data collection  **[See Methods, subheading Data set, and Results, subheading Descriptive statistics.]** |
| Participants | 6 | (*a*) Give the eligibility criteria and the sources and methods of selection of participants. Describe methods of follow-up  **[See Methods, subheading Data set.]** |
|  |  | (*b*) For matched studies, give matching criteria and number of exposed and unexposed.  **[N/A]** |
| Variables | 7 | Clearly define all outcomes, exposures, predictors, potential confounders, and effect modifiers. Give diagnostic criteria, if applicable  **[See Methods, subheadings Pre-post analysis framework, Price analyses: Random effects model, Purchase analyses: Correlated random effects tobit model, and Sensitivity analyses. Diagnostic criteria N/A.]** |
| Data sources/measurement | 8* | For each variable of interest, give sources of data and details of methods of assessment (measurement). Describe comparability of assessment methods if there is more than one group  **[See Methods, subheading Data set, sub-subheadings Nutrient profile information and tax categorization, Socioeconomic covariates, and Price indexes.]** |
| Bias | 9 | Describe any efforts to address potential sources of bias  **[We applied multiple modeling approaches to deal with potential sources of bias, and describe them in detail. See Methods, subheadings Market price aggregation, Pre-post analysis framework (which addresses the limitations of the natural experiment study design), Price analyses: Random effects model, Purchase analyses: Correlated random effects tobit model, and Sensitivity analyses.]** |
| Study size | 10 | Explain how the study size was arrived at  **[See Methods, subheading Data set, paragraph 1.]** |
| Quantitative variables | 11 | Explain how quantitative variables were handled in the analyses.  **[See Methods, subheadings Market price aggregation, Descriptive statistics, Pre-post analysis framework, Price analyses: Random effects model, Purchase analyses: Correlated random effects tobit model, and Sensitivity analyses.]** |
| Statistical methods | 12 | (*a*) Describe all statistical methods, including those used to control for confounding **[See Methods, subheadings Market price aggregation, Descriptive statistics, Pre-post analysis framework, Price analyses: Random effects model, Purchase analyses: Correlated random effects tobit model, and Sensitivity analyses.]** |
|  |  | (*b*) Describe any methods used to examine subgroups and interactions  **[We examined differences in prices and purchases by SES, noted in Methods, subheadings Price analyses: Random effects model, paragraph 2, and Purchase analyses: Correlated random effects tobit model, paragraph 1.]** |
|  |  | (*c*) Explain how missing data were addressed  **[There is not a missing data problem in the traditional sense of the word. However, the large number of zeroes found in purchasing data, whether due to infrequent purchases or due to misreporting, and how we handled this issue is discussed in Methods, subheading Purchase analyses: Correlated random effects tobit model.]** |
|  |  | (*d*) If applicable, explain how loss to follow-up was addressed  **[N/A]** |
|  |  | (*e*) Describe any sensitivity analyses.  **[See Methods, subheading Sensitivity analyses.]** |
| Results | | |
| Participants | 13* | (a) Report numbers of individuals at each stage of study—eg numbers potentially eligible, examined for eligibility, confirmed eligible, included in the study, completing follow-up, and analysed.  **[See Methods, subheading Data set, and Results, subheading Descriptive statistics.]** |
|  |  | (b) Give reasons for nonparticipation at each stage.  **[The only nonparticipation refers to households that only participated in one month during the surveyed period. Kantar WorldPanel does not provide reasons these households may have had such a short period of reporting.]** |
|  |  | (c) Consider use of a flow diagram  [**N/A]** |
| Descriptive data | 14* | (a) Give characteristics of study participants (eg demographic, clinical, social) and information on exposures and potential confounders  **[See S2 Table.]** |
|  |  | (b) Indicate number of participants with missing data for each variable of interest. **[N/A]** |
|  |  | (c) Summarise follow-up time (eg, average and total amount)  **[See Results, subheading Descriptive statistics.]** |
| Outcome data | 15* | Report numbers of outcome events or summary measures over time  **[N/A]** |
| Main results | 16 | (*a*) Give unadjusted estimates and, if applicable, confounder-adjusted estimates and their precision (eg, 95% confidence interval). Make clear which confounders were adjusted for and why they were included  **[Descriptions of confounders are in Methods, subheading Purchase analysis. Unadjusted results are in S4 Table and S5 Table. Adjusted results are in the main manuscript, Tables 1–3, and in S3 Figure, S4 Figure, and S7 Table. Tables with adjusted results include CIs. ]** |
|  |  | (*b*) Report category boundaries when continuous variables were categorized.  **[N/A]** |
|  |  | (*c*) If relevant, consider translating estimates of relative risk into absolute risk for a meaningful time period.  **[N/A]** |
| Other analyses | 17 | Report other analyses done—eg analyses of subgroups and interactions, and sensitivity analyses.  **[Subgroup results are reported in all tables. Sensitivity analyses are reported in Methods, subheading Sensitivity analyses.]** |
| Discussion | | |
| Key results | 18 | Summarise key results with reference to study objectives.  **[Main findings are reported in Discussion, subheading Main findings on H-SSB and L-SSB prices and purchases, paragraphs 1–2.]** |
| Limitations | 19 | Discuss limitations of the study, taking into account sources of potential bias or imprecision. Discuss both direction and magnitude of any potential bias.  **[Limitations and bias are discussed throughout Discussion and are explicitly addressed under subheading Strengths and limitations.]** |
| Interpretation | 20 | Give a cautious overall interpretation of results, considering objectives, limitations, multiplicity of analyses, results from similar studies, and other relevant evidence. **[Cautious interpretations are in Discussion, including direct comparisons to previous studies of SSB taxes in Mexico. See mainly subheadings Changes in prices of L-SSBs and H-SSBs after the tax and Changes in household purchases of H-SSBs and L-SSBs after the tax.]** |
| Generalisability | 21 | Discuss the generalisability (external validity) of the study results.  **[See Discussion, subheading Strengths and limitations.]** |
| Other information | | |
| Funding | 22 | Give the source of funding and the role of the funders for the present study and, if applicable, for the original study on which the present article is based  **[Funding sources are in the subsection Funding. In the acknowledgments section we note that beyond financial support, funders had no role in the study design, data collection, analysis, or interpretation.]** |

*Give information separately for exposed and unexposed groups.

Note: An Explanation and Elaboration article discusses each checklist item and gives methodological background and published examples of transparent reporting. The STROBE checklist is best used in conjunction with this article (freely available on the Web sites of PLoS Medicine at http://www.plosmedicine.org/, Annals of Internal Medicine at http://www.annals.org/, and Epidemiology at http://www.epidem.com/). Information on the STROBE Initiative is available at http://www.strobe-statement.org.
