# Supplementary material for: Chile’s 2014 sugar-sweetened beverage tax and changes in prices and purchases of sugar-sweetened beverages: An observational study in an urban environment
Source: PLoS Med. 2018 Jul 3;15(7):e1002597. doi: 10.1371/journal.pmed.1002597 (PMC6029755; doi:10.1371/journal.pmed.1002597)
Supplement: S2 Table — (PDF) [file pmed.1002597.s002.pdf]

S2 Table. Beverage categorization system

Category	Tax rate	Examples of beverage types
Untaxed	0%	plain bottled water, milk and dairy drinks, milk powders and modifiers, 100% fruit/vegetable juices, coffee, and tea
Ready-to-drink L-SSBs	10%	sodas, fruit juices, and flavored waters with less than 6.25gr/100ml
L-SSB concentrates	10%	powders and concentrates with less than 6.25gr/100ml
Noncarbonated H-SSBs	18%	fruit juices, nectars, and flavored waters with more than 6.25gr/100ml
Carbonated H-SSBs	18%	sodas with more than 6.25gr/100ml
