# Supplementary material for: Chile’s 2014 sugar-sweetened beverage tax and changes in prices and purchases of sugar-sweetened beverages: An observational study in an urban environment
Source: PLoS Med. 2018 Jul 3;15(7):e1002597. doi: 10.1371/journal.pmed.1002597 (PMC6029755; doi:10.1371/journal.pmed.1002597)
Supplement: S3 Table — (PDF) [file pmed.1002597.s003.pdf]

S3 Table. Household-level descriptive statistics (household-month averages)

	High SES<br>N = 24,756	Low SES<br>N = 39,864	Overall<br>N = 64,620	Attrition=1<br>N = 39,864
**Household region (%)**				
Santiago (region XIII)	60%	43%	47%	46%
North (regions I to IV)	8%	13%	12%	14%
Valparaiso (region V)	7%	9%	9%	9%
South-central (regions VI and VII)	7%	11%	10%	14%
Bio-Bio (region VIII)	6%	9%	9%	6%
South (regions X to XII)	11%	13%	13%	11%
**Household demographics and working status**				
Household head working (%)	68%	80%	77%	74%
Household size	3.9 (1.7)	4.3 (1.9)	4.2 (1.8)	4.0 (1.7)
Children less than 5 years old	0.2 (0.5)	0.4 (0.6)	0.3 (0.4)	0.4 (0.5)
Children between 5 and 11 years old	0.4 (0.8)	0.6 (0.8)	0.5 (0.7)	0.5 (0.7)
Children between 12 and 18 years old	0.4 (0.7)	0.5 (0.8)	0.5 (0.8)	0.5 (0.7)
Adults per household	2.8 (1.1)	2.7 (1.0)	2.8 (1.0)	2.7 (1.1)
Age of household head	53.1 (14.8)	47.7 (14.5)	49.1 (15.0)	51.6 (15.0)
**Household head education (%)**				
No formal education	3%	18%	15%	12%
Middle school	16%	38%	33%	31%
High school	31%	36%	35%	32%
College or more	50%	8%	18%	25%

Note: Weighted values using sample weights provided by Kantar WorldPanel, representative of urban households in six major zones of the country (74% of total urban population. 95% confidence intervals in parenthesis. Attrition=1 indicates households that left the survey at any point between 2013-2015.
