# Supplementary material for: Chile’s 2014 sugar-sweetened beverage tax and changes in prices and purchases of sugar-sweetened beverages: An observational study in an urban environment
Source: PLoS Med. 2018 Jul 3;15(7):e1002597. doi: 10.1371/journal.pmed.1002597 (PMC6029755; doi:10.1371/journal.pmed.1002597)
Supplement: S4 Table — (PDF) [file pmed.1002597.s004.pdf]

S4 Table. Market-level price descriptive statistics (market-month averages)

Category	Observations	Number of products	Average price (Chilean pesos)		
			Low SES	High SES	Overall
Untaxed	68,838	97	773 (766, 780)	810 (803, 817)	780 (773, 788)
Taxed 10%					
Ready-to-drink L-SSBs	16,474	23	670 (663, 673)	721 (715, 725)	684 (677,687)
L-SSB concentrates	5,363	9	158 (157, 159)	172 (171, 173)	159 (158, 160)
Taxed 18%					
Noncarbonated H-SSBs	8,271	10	870 (865, 873)	896 (891, 899)	884 (879, 886)
Carbonated H-SSBs	15,057	18	736 (731, 739)	744 (739, 748)	734 (729, 738)

Note: Weighted values using sample weights provided by Kantar WorldPanel, representative of urban households in six major zones of the country (74% of total urban population). Average and 95% CIs (in parenthesis) calculated using the Cox method .
