# Supplementary material for: Chile’s 2014 sugar-sweetened beverage tax and changes in prices and purchases of sugar-sweetened beverages: An observational study in an urban environment
Source: PLoS Med. 2018 Jul 3;15(7):e1002597. doi: 10.1371/journal.pmed.1002597 (PMC6029755; doi:10.1371/journal.pmed.1002597)
Supplement: S5 Table — (PDF) [file pmed.1002597.s005.pdf]

**S5 Table. Household level purchases descriptive statistics (household-month average)**

| Category | fraction with purchases | ml/capita/month | | | kcal/capita/month | | |
|---|---|---|---|---|---|---|---|
| | | Low SES | High SES | Overall | Low SES | High SES | Overall |
| *Untaxed* | *49%* | *12900 (13037, 12764)* | *16506 (16354, 16659)* | *13835 (13693, 13979)* | *1177.3 (1130.0, 1226.5)* | *1893.1 (1837.9, 1950.0)* | *1345.5 (1294.5, 1398.5)* |
| *Taxed 10%* | | | | | | | |
| Ready-to-drink L-SSBs | 46% | 1408 (1392, 1423) | 2591 (2563, 2619) | 1770 (1746, 1787) | 66.0 (64.7, 67.4) | 76.7 (75.0, 78.4) | 56.3 (55.5, 57.0) |
| L-SSB concentrates | 52% | 3087 (3061, 3113) | 3835 (3804, 3866) | 3245 (3218, 3245) | 11.1 (10.9, 11.3) | 5.3 (5.1, 5.4) | 9.6 (9.4, 9.8) |
| *Taxed 18%* | | | | | | | |
| Non-carbonated H-SSBs | 50% | 907 (898, 915) | 1313 (1301, 1325) | 1007 (998, 1017) | 408.1 (404.2, 412.0) | 593.0 (587.5, 598.3) | 448.7 (444.5, 453.0) |
| Carbonated H-SSBs | 74% | 3645 (3610, 3680) | 3344 (3313, 3375) | 3586 (3621, 3552) | 1504.8 (1491.5, 1518.1) | 1394.1 (1382.0, 1406.3) | 1472.9 (1459.9, 1485.9) |

Note: Weighted values using sample weights provided by Kantar WorldPanel, representative of urban households in six major zones of the country (74% of total urban population). Average and 95% CIs (in parentheses) calculated using the Cox method, considering only households with purchases in each period as pooled sample. 64,620 household monthly observations.
