# Supplementary material for: Chile’s 2014 sugar-sweetened beverage tax and changes in prices and purchases of sugar-sweetened beverages: An observational study in an urban environment
Source: PLoS Med. 2018 Jul 3;15(7):e1002597. doi: 10.1371/journal.pmed.1002597 (PMC6029755; doi:10.1371/journal.pmed.1002597)
Supplement: S6 Table — (PDF) [file pmed.1002597.s006.pdf]

S6 Table. Shapiro-Wilk normality test of log prices for selected products

Category	obs	W-stat	z-score	p-value
Untaxed				
Product ID 5271	643	0.94	-1.15	0.88
Taxed 10%				
Product ID 5221	683	0.90	-1.15	0.87
Product ID 4881	856	0.86	-0.97	0.83
Taxed 18%				
Product ID 5312	863	0.88	-1.11	0.87
Product ID 5172	821	0.96	-1.12	0.87

Note: Product IDs were coded and selected randomly. Shapiro-Wilk test has a null hypotesis of normality. W-stat indicates the Shapiro-Wilk statistic.
