# Supplementary material for: Chile’s 2014 sugar-sweetened beverage tax and changes in prices and purchases of sugar-sweetened beverages: An observational study in an urban environment
Source: PLoS Med. 2018 Jul 3;15(7):e1002597. doi: 10.1371/journal.pmed.1002597 (PMC6029755; doi:10.1371/journal.pmed.1002597)
Supplement: S7 Table — (PDF) [file pmed.1002597.s007.pdf]

S7 Table. Average adjusted real prices, actual versus counterfactual estimates by brand market share and package size (Chilean pesos)

Category	Absolute difference				% change		Package size	% change		Brand size
	Small package	Large package	Low market share	High market share	Small package	Large package	(p-value)	Low market share	High market share	(p-value)
Untaxed	**46.3 (29.7, 62.9)**	-4.6 (-9.9, 0.7)	**14.9 (5.9, 24.0)**	9.4 (-0.4, 19.2)	**6.4% (4.1%, 8.7%)**	-0.6% (-1.3%, 0.1%)	0.000	**2.0% (0.8%, 3.2%)**	1.4% (-0.1%, 2.9%)	0.350
Taxed 10%										
Ready-to-drink L-SSBs	**37.0 (14.3, 59.7)**	1.7 (-3.7, 7.2)	*8.5 (-1.5, 18.4)*	16.5 (5.2, 27.8)	**2.9% (1.1%, 4.7%)**	0.3% (-0.7%, 1.3%)	0.018	1.1% (-0.2%, 2.4%)	2.7% (0.9%, 4.5%)	0.182
L-SSB concentrates	**-69.1 (-73.5, -64.7)**	-3.7 (-7.6, 0.2)	**-26.1 (-30.2, -21.9)**	**11.1 (7.2, 15.1)**	**-40.0% (-42.6%, -37.4%)**	-2.6% (-5.3%, 0.1%)	0.000	**-1.6% (-1.9%, -1.3%)**	**7.4% (4.8%, 10.1%)**	0.000
Taxed 18%										
Noncarbonated H-SSBs	**62.1 (33.0, 91.2)**	6.7 (-6.1, 19.6)	**35.4 (15.3, 55.5)**	23.6 (3.3, 43.9)	**5.5% (2.9%, 8.1%)**	1.1% (-1.0%, 3.2%)	0.005	**4.0% (1.7%, 6.3%)**	2.9% (0.4%, 5.4%)	0.431
Carbonated H-SSBs	**29.9 (13.4, 46.4)**	**9.9 (3.8, 16.0)**	**14.5 (7.8, 21.1)**	**14.6 (5.3, 23.9)**	**2.2% (1.0%, 3.4%)**	**1.9% (0.7%, 3.0%)**	0.018	**1.9% (1.0%, 2.8%)**	**2.0% (0.7%, 3.3%)**	0.986

Note: Small packages coded as less than or equal to 2,500mL and large package otherwise. Small brands coded as less than or equal to 10% to total sales, and large brands othewise. 95% CIs in parentheses. Results significantly different from zero indicated in bold, using the Sidak-Dunn correction for multiple hypothesis testing (critical value alpha = 0.0102). Unweighted estimates. Package size and brand size (p-value) indicates the difference in means test between the two groups for the predicted absolute differences. Observations in each model as indicated in Table 4.
