# Supplementary material for: Chile’s 2014 sugar-sweetened beverage tax and changes in prices and purchases of sugar-sweetened beverages: An observational study in an urban environment
Source: PLoS Med. 2018 Jul 3;15(7):e1002597. doi: 10.1371/journal.pmed.1002597 (PMC6029755; doi:10.1371/journal.pmed.1002597)
Supplement: S8 Table — (PDF) [file pmed.1002597.s008.pdf]

S8 Table. Alternative model specifications for selected categories

	Prices							
	Absolute difference (versus counterfactual)				R-squared			
**Model specification**	Ready-to-drink L-SSBs	L-SSB concentrates	Noncarbonated H-SSBs	Carbonated H-SSBs	Ready-to-drink L-SSBs	L-SSB concentrates	Non-carbonated H-SSBs	Carbonated H-SSBs
Quadratic time trends and quarterly seasonality	11.1 (2.2 , 20.0)	-11.0 (-13.6 , -7.6)	33.3 (13.9 , 52.7)	14.7 (7.5 , 21.9)	0.525	0.554	0.781	0.882
Arellano-Bond lagged model	4.1 (-4.8, 12.3)	-13.5 (-17.1, -9.8)	4.4 (-7.1, 15.9)	1.9 (-4.4, 8.1)	-	-	-	-
Monthly fixed effects	-0.3 (-7.1, 6.5)	-21.6 (-21.8, -19.5)	4.9 (-4.3, 14.2)	4.6 (-0.5, 9.7)	0.522	0.548	0.776	0.878
Break in intercept and trends (quadratic)	71.1 (-1.5, 145)	25.7 (-15.0, 66.4)	108.7 (20.3, 197.0)	110.2 (37.4 182.9)	0.523	0.551	0.776	0.881
Alternative time break (April 2014)	8.3 (-1.9, 18.6)	-1.1 (-4.3, 2.2)	15.0 (-1.1, 31.1)	20.4 (11.9 28.9)	0.520	0.542	0.774	0.877

	Quantities (volume)							
	Absolute difference (versus counterfactual)				Akaike information criterion			
**Model specification**	Ready-to-drink L-SSBs	L-SSB concentrates	Noncarbonated H- SSBs	Carbonated H-SSBs	Ready-to-drink L-SSBs	L-SSB concentrates	Non-carbonated H-SSBs	Carbonated H-SSBs
Panel Tobit with correlated random effects	123 (84 , 162)	165 (105 , 225)	-41 (-68, -15)	-70 (-142 , 2)	621,029	712,458	624,792	979,654
Static hurdle model	94 (38, 151)	112 (35, 188)	-19 (-46, 8)	-14 (-124, 97)	-	-	-	-
Break in intercept and trends (quadratic)	-19 (-291, 252)	-413 (-709, -118)	-42 (-215, 130)	760 (141, 1,379)	618,566	714,424	624,793	979,662
Alternative time break (April 2014)	43 (3, 83)	50 (-11, 112)	7 (-16, 31)	-257 (-596, 81)	619,316	714,448	624,802	979,684

For each model, AIC was calculated using the estimated log-likelihood. Model descriptions as follows. Quadratic trends and quarterly seasonality is the preferred specification for prices. Panel Tobit with correlated random effects is the preferred specification for quantities (volume and kcal). Arellano-Bond consistently estimates a model with a lagged depedent variable (R-squared is not defined in dynamic model). Monthly fixed-effects replaces time trends and seasonality with month-by-month indicator variables. Break in intercept and trends allows for different pre and post trends after implementation. Static hurdle model relaxes Tobit specification of common parameters to characterize the censored distribution (AIC not comparable since model is not nested). Alternative time-break tests whether there is significance in a period previous to the implementation of the law (exactly when the bill was introduced to congress.
