# Supplementary figures and images for: Chile’s 2014 sugar-sweetened beverage tax and changes in prices and purchases of sugar-sweetened beverages: An observational study in an urban environment
Source: PLoS Med. 2018 Jul 3;15(7):e1002597. doi: 10.1371/journal.pmed.1002597 (PMC6029755; doi:10.1371/journal.pmed.1002597)

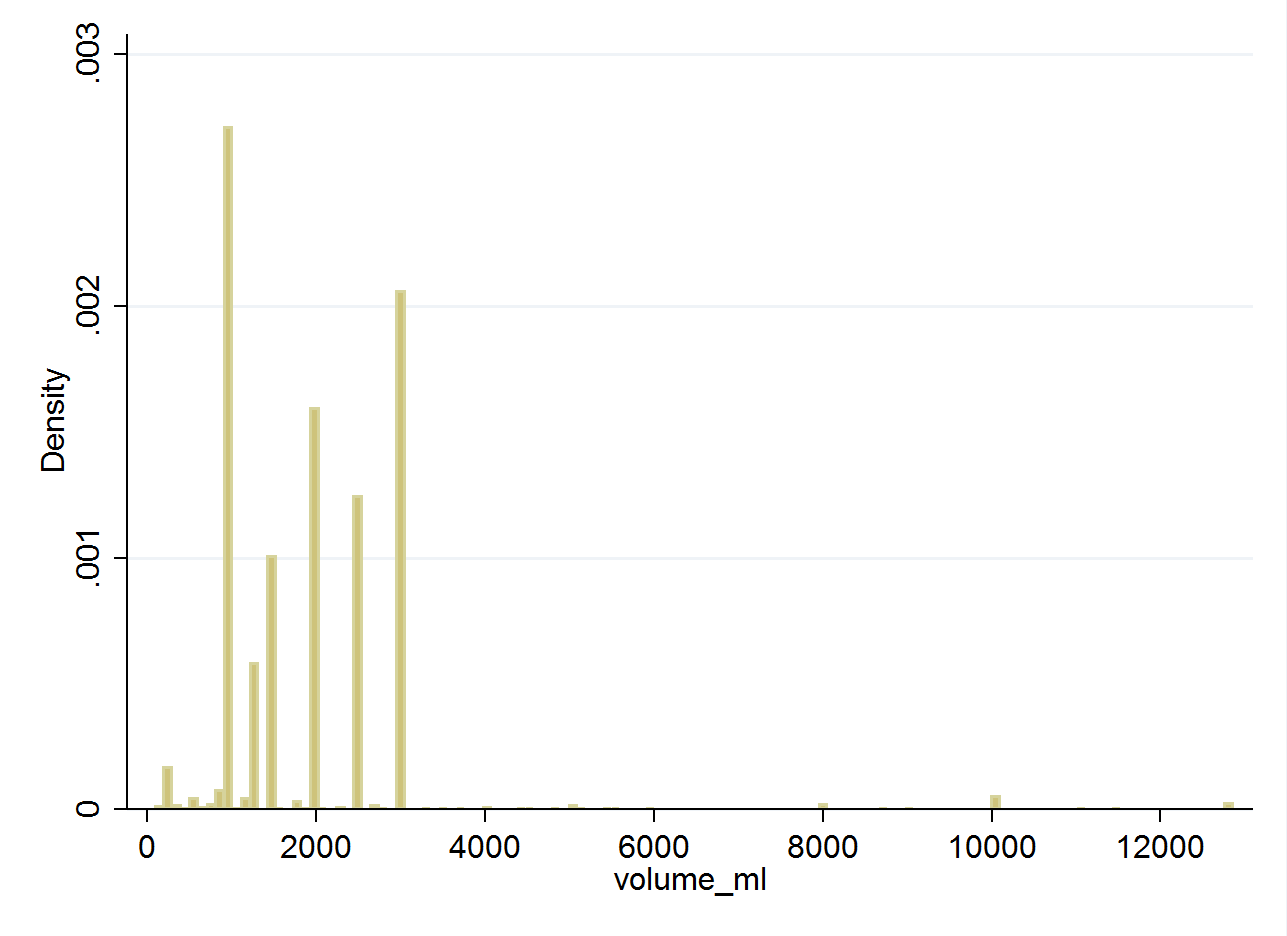

Supplement: S1 Fig — (TIF) [file pmed.1002597.s009.tif]

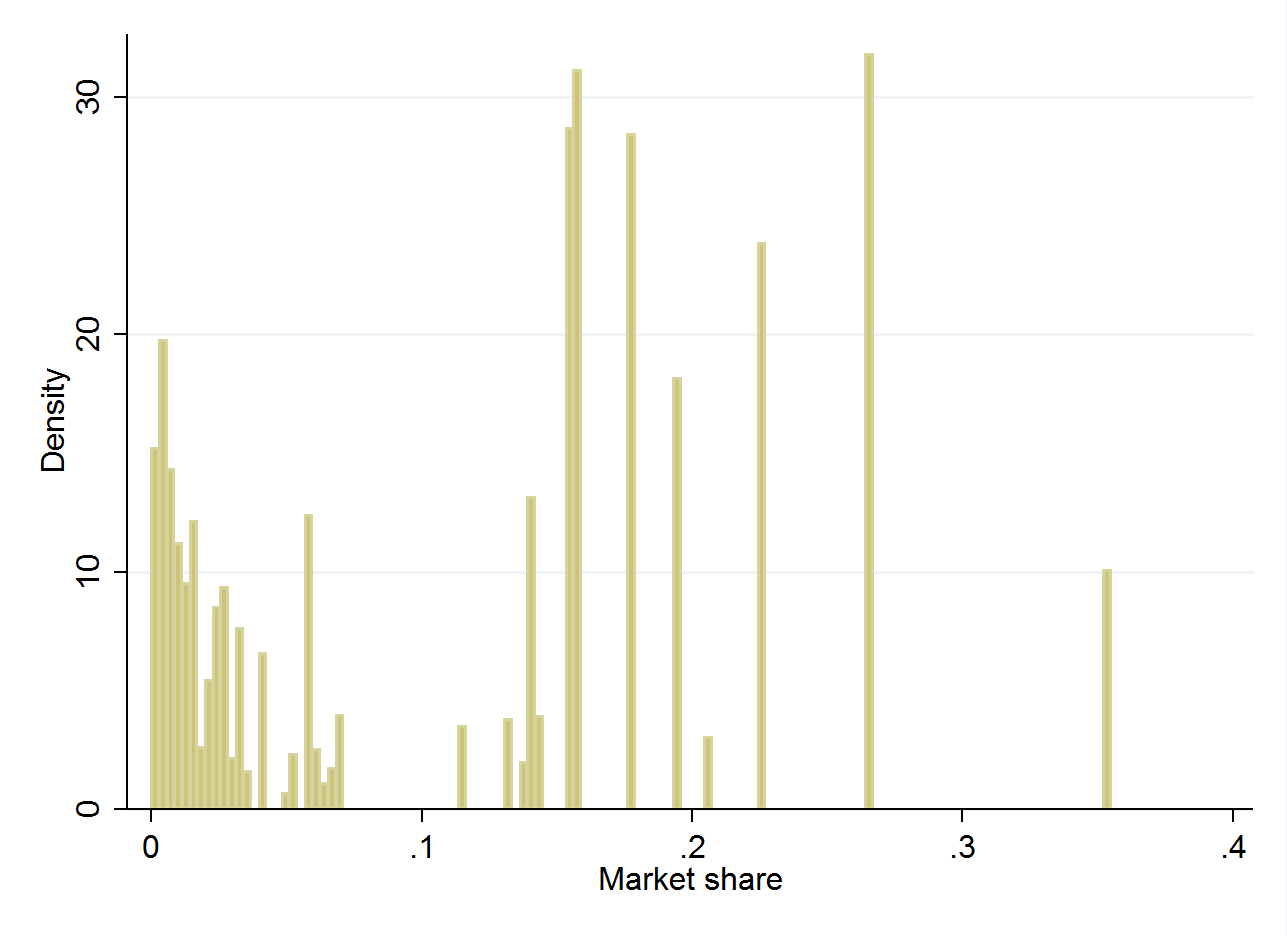

Supplement: S2 Fig — (TIF) [file pmed.1002597.s010.tif]

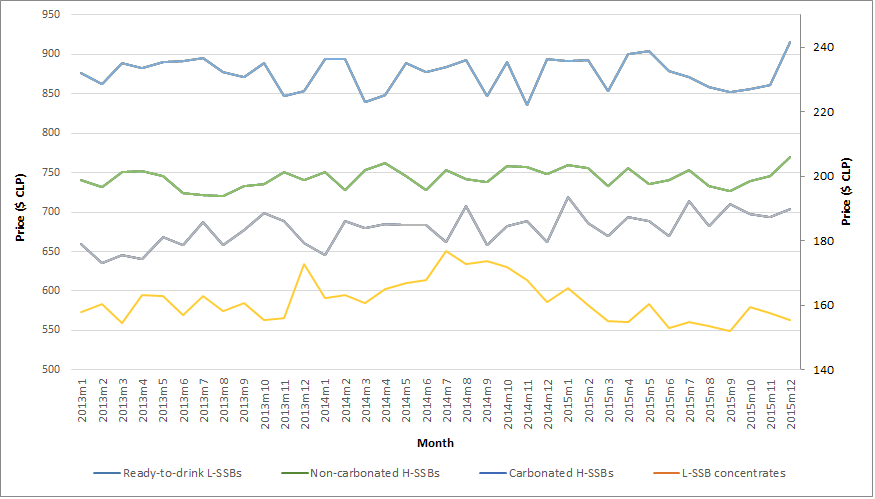

Supplement: S3 Fig — H-SSBs and ready-to-drink L-SSBs on left axis, L-SSB concentrates on right axis. H-SSB, industrialized beverage with high levels of sugar; L-SSB, industrialized beverage with low or no sugar. (TIF) [file pmed.1002597.s011.tif]

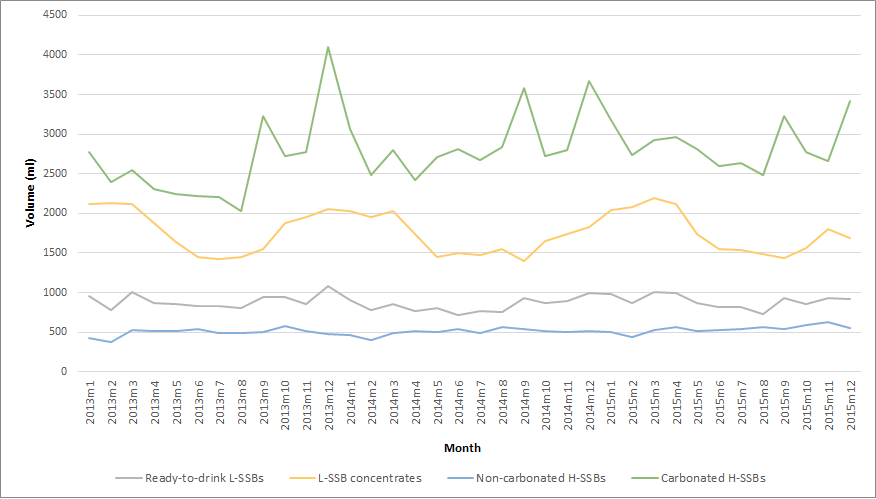

Supplement: S4 Fig — (TIF) [file pmed.1002597.s012.tif]
